# Characterization of neutralizing epitopes in antigenic site B of recently circulating influenza A(H3N2) viruses

**DOI:** 10.1099/jgv.0.001101

**Published:** 2018-06-26

**Authors:** Kerstin Beer, Mian Dai, Steven Howell, Pramila Rijal, Alain R. Townsend, Yipu Lin, Stephen A. Wharton, Rodney S. Daniels, John W. McCauley

**Affiliations:** ^1^​ Crick Worldwide Influenza Centre, The Francis Crick Institute, 1 Midland Road, London NW1 1AT, UK; ^2^​ Mass Spectrometry and Proteomics Laboratory, The Francis Crick Institute, 1 Midland Road, London NW1 1AT, UK; ^3^​ MRC Human Immunology Unit, Weatherall Institute of Molecular Medicine, University of Oxford, John Radcliffe Hospital, Oxford OX3 9DS, UK

**Keywords:** Influenza, antigenic drift, monoclonal antibodies

## Abstract

Influenza A(H3N2) viruses are associated with outbreaks worldwide and can cause disease with severe complications. The impact can be reduced by vaccination, which induces neutralizing antibodies that mainly target the haemagglutinin glycoprotein (HA). In this study we generated neutralizing mouse monoclonal antibodies (mAbs) against A/Victoria/361/2011 and identified their epitopes by generating and sequencing escape viruses. The epitopes are located in antigenic site B, which is near the receptor-binding site and is immunodominant in humans. Amino acid (aa) substitutions at positions 156, 158, 159, 189, 190 and 193 in antigenic site B led to reduced ability of mAbs to block receptor-binding. The majority of A(H3N2) viruses that have been circulating since 2014 are antigenically distinct from previous A(H3N2) viruses. The neutralization-sensitive epitopes in antigenic site B of currently circulating viruses were examined with these mAbs. We found that clade 3C.2a viruses, possessing an additional potential glycosylation site at HA1 position N158, were poorly recognized by some of the mAbs, but other residues, notably at position 159, also affected antibody binding. Through a mass spectrometric (MS) analysis of HA, the glycosylated sites of HA1 were established and we determined that residue 158 of HA1 was glycosylated and so modified a neutralization-sensitive epitope. Understanding and monitoring individual epitopes is likely to improve vaccine strain selection.

## Introduction

Influenza A(H3N2) viruses are associated with outbreaks worldwide and can cause disease with severe complications. The infection is preventable by vaccination, which induces neutralizing antibodies that mainly target the haemagglutinin glycoprotein (HA) [1]. The HA is embedded in the virus membrane as a homotrimer in which each monomer is composed of two disulphide-linked polypeptides, HA1 and HA2. HA1 forms a globular head domain containing the receptor-binding site that targets sialic acid residues on host cells, whereas HA2 contains the transmembrane anchor domain (C-terminal) and a fusion domain (N-terminal) and forms a stem-like structure that undergoes a large conformational change at the low pH of the endosome to mediate fusion of the virus envelope and the endosomal membrane [1].

The vast majority of neutralizing antibodies induced by infection or vaccination are directed against the exposed and highly variable loops surrounding the receptor-binding site in HA1 and prevent the attachment of virus to cell receptors [2–5]. Five distinct antigenic sites (A–E) have been identified by sequencing the HA gene of viruses selected for resistance to monoclonal antibodies (mAbs) made in mice and defined by determining amino acid (aa) substitutions in circulating viruses [2, 3, 6–8]. However, the antibody response can be biased towards a limited number of immunodominant neutralization-sensitive epitopes. It has been postulated that the human and ferret antibody responses are focused on antigenic sites A and B [9–13]. Using human antisera after vaccination in the 2006–07 and/or 2008–09 seasons, Popova *et al*. concluded that antigenic site B is immunodominant over antigenic site A [14]. Both sites A and B are located on the top of HA1 flanking the receptor-binding pocket [8]. Koel *et al*. investigated aa substitutions that emerged in human A(H3N2) viruses between 1968 and 2003 to identify critical residues associated with major antigenic change and identified the aa residue at position 145 (antigenic site A) and residues at positions 155, 156, 158, 159, 189 and 193 (antigenic site B) as being primarily responsible for antigenic drift [15].

The majority of A(H3N2) viruses that have been circulating since 2014 are known to be antigenically different from previously circulating viruses and can be grouped into three genetically distinct clades: 3C.2a, 3C.3a and 3C.3b [16, 17]. The HAs of viruses in these clades have specific aa changes in antigenic site B: 3C.3a – F159S; 3C.3b – L157S; 3C.2a – F159Y and K160T. The change K160T resulted in a new potential glycosylation site at N158. The number of N-linked sequons (Asn-X-Ser/Thr) in the HA has increased over the course of the evolution of A(H3N2) viruses and these additional oligosaccharide chains can contribute to immune evasion [1, 9, 18–22].

In this study a panel of mAbs generated against influenza A(H3N2) viruses from 2011 has been used to compare recently circulating A(H3N2) viruses with earlier A(H3N2) viruses to identify aa substitutions affecting antibody recognition. These mAbs were directed against distinct but overlapping epitopes in antigenic site B. We identified aa substitutions in HA of circulating viruses affecting these neutralization-sensitive epitopes and have established how recognition by these antibodies is affected by the glycosylation of site B in recently circulating A(H3N2) viruses.

## Results

### Generation of neutralizing mAbs against HA and localisation of epitopes

A panel of neutralizing mouse mAbs against the HA of A(H3N2) virus A/Victoria/361/2011 (Vic361), propagated in MDCK-SIAT1 cells [Vic361(c)] or in embryonated hens’ eggs [Vic361(e)], was generated by hybridoma technology. Vic361(e) possessed two aa substitutions in HA1 – H156R and G186V – compared to Vic361(c). The generated neutralizing mAbs, 7-1-3, 11-2-6, 16-3-5, 17-1-1 and 24-3-5, were induced by Vic361(c), whereas mAbs 100-1-1 and 101-1-1 were induced by Vic361(e).

Antibody escape viruses were generated to localize the corresponding neutralization-sensitive epitopes. Vic361(e) was propagated in the presence of mAb in embryonated hens’ eggs. After two or three passages, haemagglutination inhibition (HI) assays with the corresponding mAbs were performed to demonstrate antibody escape and the HA genes from these viruses were sequenced (Table 1). All escape viruses carried aa substitutions in HA1. Viruses obtained after propagation in the presence of mAb 7-1-3 showed D190E in combination with either K189N or F193L. Viruses obtained in the presence of mAb 11-2-6 showed variously, F193S, D190E combined with N158D, or D190E combined with F159S. mAb 16-3-5 induced escape viruses possessing D190E and F193S/V or K189Q with D190E. Escape viruses obtained in the presence of mAb 17-1-1 possessed N158D with D190E. Escape viruses with F193S were induced by mAbs 24-3-5 and 100-1-1. mAb 100-1-1 also induced viruses possessing R156C. Escape viruses obtained after propagation in the presence of mAb 101-1-1 showed aa substitution F159S. It was striking that escape variants were frequently identified with two aa substitutions, most notably with the substitution D190E for variants selected with antibodies raised against Vic361(c).

**Table 1.** aa substitutions in haemagglutinin of antibody escape viruses

mAb	aa substitutions	Virus used for immunization
7-1-3	D190E and F193L; K189N and D190E	Vic361(c)
11-2-6	F193S; N158D and D190E; F159S and D190E	Vic361(c)
16-3-5	D190E and F193V; D190E and F193S; K189Q and D190E	Vic361(c)
17-1-1	D190E and N158D	Vic361(c)
24-3-5	F193S	Vic361(c)
100-1-1	F193S; R156C	Vic361(e)
101-1-1	F159S	Vic361(e)

To further define the substitutions affecting the ability of mAbs to block receptor-binding, mutations engineered into a cDNA encoding the HA of Vic361(c) were used to generate reverse genetics (RG)-derived viruses possessing aa changes identified in mAb-resistant variants, which were then tested against mAbs in HI assays (Table 2). These RG viruses had HA and neuraminidase (NA) genes from Vic361(c) and all other genes from A/Puerto Rico/8/34.

**Table 2.** Concentration of mAbs (ng ml^−1^) required to inhibit haemagglutination by RG viruses

mAb induced by:	Vic361(c)					Vic361(e)	
Virus	mAb					mAb	
	7-1-3	11-2-6	16-3-5	17-1-1	24-3-5	100-1-1	101-1-1
Vic361(e)	39	19.5	39	19.5	19.5	625	625
RG/Vic361 R156C	39	**625**	78	**312.5**	**1250**	**>10 000**	**>10 000**
RG/Vic361 N158D	19.5	**2500**	39	**2500**	**625**	**10 000**	625
RG/Vic361 F159S	78	**1250**	78	**1250**	**625**	**>10 000**	**>10 000**
RG/Vic361 K189Q	**1250**	19.5	**10 000**	19.5	39	1250	312.5
RG/Vic361 K189N	**1250**	19.5	**10 000**	19.5	39	2500	312.5
RG/Vic361 D190E	78	**625**	78	156.25	**1250**	1250	2500
RG/Vic361 F193L	156.25	**>10 000**	**1250**	**>10 000**	**>10 000**	**>10 000**	**10 000**
RG/Vic361 F193S	**5000**	**>10 000**	**>10 000**	**>10 000**	**>10 000**	**>10 000**	625
RG/Vic361 F193V	**1250**	**>10 000**	**10 000**	**10 000**	**10 000**	**>10 000**	312.5
RG/Vic361 D190E+F193L	**>10 000**	**>10 000**	**>10 000**	**>10 000**	**>10 000**	**>10 000**	**10 000**
RG/Vic361 D190E+F193S	**>10 000**	**>10 000**	**>10 000**	**>10 000**	**>10 000**	**>10 000**	1250
RG/Vic361 D190E+F193V	**>10 000**	**>10 000**	**>10 000**	**>10 000**	**>10 000**	**>10 000**	1250

Values highlighted in bold indicate where the concentrations of antibody needed to inhibit haemagglutination were at least 10 times higher than that needed for Vic361(e) or Vic361(c).

aa substitutions R156C, N158D and F159S, as well as D190E and/or F193L/S/V, decreased the ability of mAbs 11-2-6, 17-1-1 and 24-3-5 to block receptor-binding. The substitutions K189Q/N and F193S/V decreased the ability of mAbs 7-1-3 and 16-3-5 to block receptor-binding with D190E, which was identified in the neutralization-resistant variant viruses selected with mAbs 7-1-3 and 16-3-5, being incidental to the antigenicity of the selected variants. F193L additionally reduced the ability of mAbs 7-1-3 and 16-3-5 to inhibit virus haemagglutination, but had less impact than the F193S and F193V substitutions. Although D190E did not show an effect on the ability to block haemagglutination by mAbs 7-1-3 and 16-3-5, in combination with F193L/S/V it prevented the blocking of the receptor-binding of virus by these mAbs. Whether D190E and F193S act together to circumvent inhibition by mAbs 7-1-3 and 16-3-5 is less certain- because the differences in antibody recognition between the F193S variant and the double-substitution variant were in the order of twofold for mAb 7-1-3 and virus with the F193S substitution only was fully resistant to mAb 16-3-5 in the HI assay. mAbs 11-2-6, 17-1-1 and 24-3-5 were also unable to block haemagglutination by viruses with substitutions at position 193 of HA1 and, additionally, failed to inhibit haemagglutination of viruses with substitutions R156C, N158D and F159S. The substitutions R156C, N158D, F159S and F193L/S/V also affected the ability of mAb 100-1-1, raised against Vic361(e), to block receptor-binding, and the ability of mAb 101-1-1 to block receptor-binding was reduced in viruses with the engineered R156C, F159S and F193L substitutions, representing a subset of those that were less sensitive to inhibition by mAb100-1-1.

**Fig. 1.**
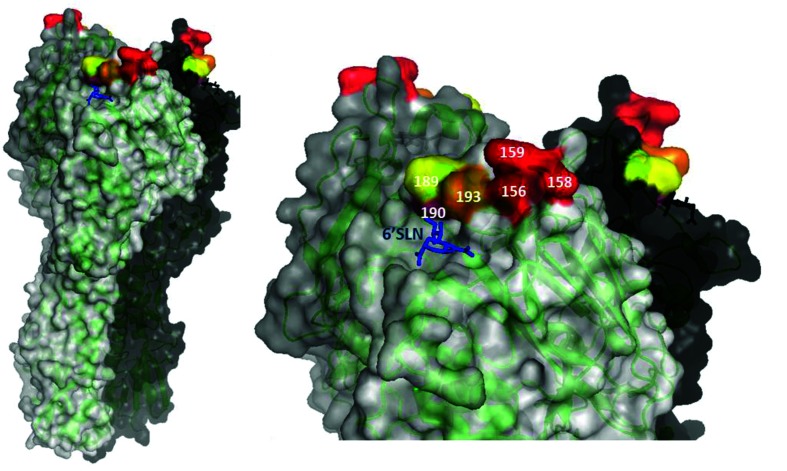
Critical residues in antigenic site B neutralization-sensitive epitopes of Vic361 HA. The adapted RCSB PDB file 4WEA [41] shows HA monomers in black, white and grey. Critical aa positions are highlighted. Positions 156, 158, 159 (red), 190 (purple) and 193 (orange) are critical for mAbs 11-2-6, 17-1-1 and 24-3-5; positions 189 (yellow) and 193 (orange) are critical for mAbs 7-1-3 and 16-3-5; positions 156, 158, 159 (red) and 193 (orange) are critical for 100-1-1; positions 156, 159 (red) and 193 (orange) are critical for 101-1-1. Blue, sialic acid (6′SLN) in the receptor-binding site. The figures were generated with PyMOL.

The identified aa substitutions represent critical residues in the neutralization-sensitive epitopes of the generated mAbs. These residues were all located in antigenic site B, near the receptor-binding site (Fig. 1).

### Inhibition of haemagglutination by earlier circulating A(H3N2) viruses by mAbs

Several of the aa changes found in the HA of our antibody escape viruses had been reported in earlier circulating viruses. The aa residue S189 changed to N189 in 2003/2004 [23] and to K189 in 2009 [24]. The change E190D occurred in viruses isolated from 1991 and affects the receptor-binding properties of influenza viruses [25]. Viruses isolated after 2005 possessed S193F, which showed a negligible effect on receptor-binding [26], and changes at position 156 are often related to virus adaptation during propagation in embryonated hens’ eggs [27, 28].

HI assays were carried out to test the ability of our mAbs to block agglutination of red blood cells (RBCs) by earlier circulating viruses possessing changes at these positions in antigenic site B. The mAbs were able to block the receptor-binding of Vic361 and A/Perth/16/2009 viruses, but not the earlier A/Brisbane/10/2007, A/Wyoming/03/2003 and A/Moscow/10/99 viruses (Table 3). These latter viruses have different aa residues in antigenic site B that affect the epitopes recognized by neutralizing mAbs.

**Table 3.** Concentration of mAb (ng ml^−1^) required to inhibit haemagglutination by earlier A(H3N2) viruses

Virus	Year isolated	aa						mAb				
		156	158	160	189	190	193	7-1-3	11-2-6	16-3-5	17-1-1	24-3-5
Vic361(c)	2011	H	N	K	K	D	F	78	39	39	39	39
A/Perth/16/2009	2009	H	N	K	K	D	F	156.25	39	156.25	78	78
A/Brisbane/10/2007	2007	H	**K**	K	**N**	D	F	**10 000**	**1250**	**5000**	**5000**	**5000**
A/Wyoming/03/2003	2003	H	**K**	K	**S**	D	**S**	**>10 000**	**>10 000**	**>10 000**	**>10 000**	**>10 000**
A/Moscow/10/99	1999	**Q**	**K**	**R**	**S**	D	**S**	**>10 000**	**>10 000**	**>10 000**	**>10 000**	**>10 000**

aa that are different to those in Vic361(c) are highlighted in bold. Concentrations (ng ml^−1^) of mAbs that showed a ≥10-fold increase in comparison to Vic361(c) are highlighted in bold.

### Neutralization of recently circulating A(H3N2) viruses by mAbs

In 2014 new genetic clades of A(H3N2) viruses emerged: 3C.2a, 3C.3a and 3C3b [17, 29, 30]. Amongst others, these viruses show aa substitutions in antigenic site B of the HA; most notably, 3C.3a viruses possess F159S, 3C.3b viruses possess L157S, and most 3C.2a viruses possess F159Y and K160T. Importantly, the substitution K160T leads to a new potential glycosylation motif at residues 158 to 160 in HA1, which is associated with an inability to agglutinate guinea pig RBCs such that viruses cannot be analysed by HI assays [31].

To determine the effect of the changes regarding the neutralizing ability of our mAbs, we tested the 3C.3a virus A/Switzerland/9715293/2013 (Switz13), the 3C.3b virus A/Bucuresti/17949/2015 and the 3C.2a virus A/Israel/O-7414/2014 in HI assays. A/Israel/O-7414/2014, with HA1 160K, lacked the new glycosylation motif at residues 158–160 and was able to agglutinate guinea pig RBCs and be analysed by HI.

The panel of generated mAbs was able to block the receptor-binding of Vic361(c), A/Israel/O-7414/2014 and A/Bucuresti/17949/2015. However, to block haemagglutination of Switz13, higher concentrations of each of the mAbs were needed (Table 4).

**Table 4.** Concentration of mAb (ng ml^−1^) required to prevent haemagglutination or virus infection by recently circulating A(H3N2) and RG viruses

Virus	clade	aa				mAb				
		157	158	159	160	7-1-3	11-2-6	16-3-5	17-1-1	24-3-5
HI results										
Vic361(c)	3C.1	L	N	F	K	78	39	39	39	39
A/Israel/O-7414/2014(c)	3C.2a	L	N	**Y**	K	39	19.5	39	39	39
Switz13(c)	3C.3a	L	N	**S**	K	312.5	312.5	312.5	156.25	625
A/Bucuresti/17949/2015(c)	3C.3b	**S**	N	F	K	78	39	78	39	39
MN results										
Vic361(c)	3C.1	L	N	F	K	<39	<39	<39	nd	<39
A/Israel/O-7414/2014(c)	3C.2a	L	N	**Y**	K	39	<39	39	nd	<39
A/Lisboa/SU63/2014(c)	3C.2a	L	N	**Y**	**T**	156.25	**>5000**	156.25	nd	**>5000**
RG/Vic361(c) F159Y		L	N	**Y**	K	<39	<39	<39	nd	<39
RG/Vic361(c) F159Y+K160T		L	N	**Y**	**T**	156.25	**>5000**	156.25	nd	**>5000**

The concentrations of mAbs (ng ml^−1^) necessary to inhibit agglutination of the virus by HI or to reduce the number of plaques by 50 % (PRNA) are shown. nd, not done.

To determine whether the new potential glycosylation site at N158 of 3C.2a viruses affects the epitopes of the generated mAbs, a microneutralization (MN) assay was performed with the 3C.2a viruses A/Lisboa/SU63/2014 and A/Israel/O-7414/2014, and the 3C.1 virus Vic361(c) (Table 4). A/Lisboa/SU63/2014 possessed F159Y and K160T, encoding the acquired glycosylation motif at residues 158–160 of HA1, and therefore could not be analysed by HI. Additionally, RG/Vic361(c) viruses that possessed either F159Y or F159Y and K160T in HA1 were also tested. mAbs 7-1-3, 11-2-6, 16-3-5 and 24-3-5 were able to neutralize Vic361(c), as well as A/Israel/O-7414/2014 (F159Y, 160K); in contrast, A/Lisboa/SU63/2014 (F159Y, K160T) was only neutralized by mAbs 7-1-3 and 16-3-5. Similarly, RG/Vic361(c) F159Y+K160T, engineered to encode the glycosylation motif at residues 158–160 in HA1, was only neutralized by mAbs 7-1-3 and 16-3-5.

### Neutralization of RG viruses by mAbs

The RG viruses were made based on a clade 3C.3a virus (A/Finland/438/2014) and a clade 3C.2a virus (A/Hong Kong/4800/2014), both propagated solely in cell culture, and variant viruses were made that differed at residues 158 to 160 in HA1 to further refine the specificity of the mAbs. Neutralization was assessed by MN assays (Table 5). These RG viruses had the HA gene from the appropriate H3 virus and all other genes from A/WSN/33.

**Table 5.** Concentration of mAb (ng ml^−1^) required to neutralize wild-type and RG viruses

Test virus	Clade	aa			mAb (ng ml^−1^) for 50 % inhibition				
		158	159	160	7-1-3	16-3-5	11-2-6	17-1-1	24-3-5
WT-A/Victoria/361/2011 (Vic361(c))	3C.1	N	F	K	<39	<39	<39	<39	<39
RG-A/Victoria/361/2011 (HA+NA/PR8)		N	F	K	<39	<39	<39	<39	<39
WT-A/Finland/438/2014	3C.3a	N	**S**	K	156	156	1250	625	2500
RG-A/Finland/438/2014 (HA/WSN)		N	**S**	K	312	156	1250	1250	1250
RG-A/Finland438/2014 (HAS159F/WSN)		N	F	K	156	78	39	39	<39
RG-A/Finland438/2014 (HAS159F+K160T/WSN)		N	F	**T**	625	625	**>5000**	**>5000**	**>5000**
WT-A/Hong Kong/4800/2014	3C.2a	N	**Y**	**T**	1250	1250	**>5000**	**>5000**	**>5000**
RG-A/Hong Kong/4800/2014 (HA/WSN)		N	**Y**	**T**	625	312	**>5000**	**>5000**	**>5000**
RG-A/Hong Kong/4800/2014 (HA Y159F/WSN)		N	F	**T**	156	78	**5000**	**5000**	**5000**
RG-A/Hong Kong/4800/2014 (HA T160K/WSN)		N	**Y**	K	78	39	<39	<39	<39
RG-A/Hong Kong/4800/2014 (HA Y159F+T160K/WSN)		N	F	K	39	<39	<39	<39	<39

The concentrations of mAbs (ng ml^−1^) necessary to reduce the number of plaques by 50 % (PRNA) are shown; values in bold indicate where the concentration was ≥10 times higher than that needed for Vic361(c).

A/Finland/438/2014 and the corresponding RG virus showed low-level neutralization by the mAbs, but introduction of the HA1 S159F substitution, reverting the substitution in the HA of clade 3C.3a viruses to that of clade 3C.1 viruses, restored highly effective neutralization by four of the five mAbs. The introduction of a glycosylation motif at residues 158–160 of HA1 eliminated the binding of three of the mAbs (11-2-6, 17-1-1 and 24-3-5) and reduced binding of the other two mAbs (7-1-3 and 16-3-5). In the RG viruses based on the clade 3C.2a virus, the presence of the glycosylation motif at residues 158–160 of HA1, as expected, eliminated binding by mAbs 11-2-6, 17-1-1 and 24-3-5, and reduced binding of mAbs 7-1-3 and 16-3-5. It was, however, notable that 3C.2a viruses with either 159F or 159Y were recognized by mAbs 7-1-3 and 16-3-5 at titres within twofold of the titres with Vic361(c).

Thus, antibodies 11-2-6, 17-1-1 and 24-3-5 failed to neutralize viruses when the glycosylation motif was present, whether in a natural isolate or one derived by RG, and the substitutions at residue F159Y/S also affected virus neutralization by a subset of the antibodies.

### Utilization of N-linked glycosylation at position 158 in HA of a clade 3C.2a virus

**Fig. 2.**
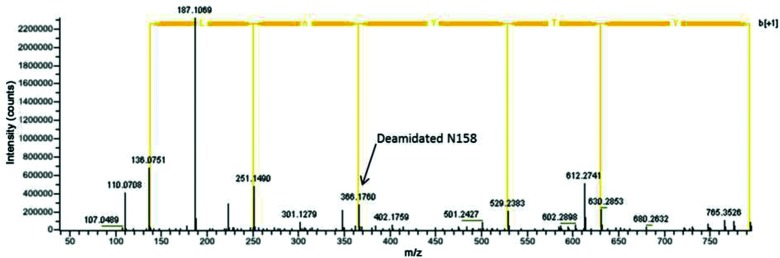
MS/MS spectrum for deamidated peptides containing glycosylation site NYT at positions 158–160 of HA1. The aa sequence is shown for the b ion series. The y series is not shown for simplicity. Peak 187.1069 equals the y2 ion. The position of the deamidated N158 is indicated.

Most influenza viruses of clade 3C.2a possess the new potential glycosylation motif NYT at position 158–160, located on the top of antigenic site B. To determine definitively whether this motif directs glycosylation of N158 of HA1, the HA of virus A/Lisboa/SU63/2014 (3C.2a) was purified and digested with PNGase F to remove N-glycans and the deamidated motifs were then identified by mass spectrometry (MS) as markers of glycosylation (Fig 2).

The background deamidation, determined by the ratio of asparagine deamidation to amidation when asparagine was not in a glycosylation motif, was found to be very low, typically 1 %. The determination of highly deamidated asparagine residues identified 11 N-linked glycosylation sites on the HA1 of A/Lisboa/SU63/2014: N^8^ST, N^22^GT, N^38^AT, N^45^SS, N^63^CT, N^126^WT, N^133^GT, N^158^YT, N^165^VT, N^246^ST and N^285^GS. There was no evidence of glycosylation on site N^122^ES. The potential glycosylation site N^483^GT, located in HA2, was not recovered. As a control, MS was similarly performed on A/Israel/O-7414/2014 that lacked a glycosylation sequon at residue 158–160. There was no evidence of deamidation at N^158^YK (not shown).

Our results confirmed biochemically that NYT at position 158 of HA1 of 3C.2a viruses is glycosylated and indicate that this additional N-linked carbohydrate chain is likely to modify the epitopes of mAbs 11-2-6 and 24-3-5 so that the antibodies can no longer neutralize the virus.

### Ferret and human antisera recognising neutralization-sensitive epitopes in antigenic site B

Post-infection ferret antisera are used to assess the antigenic properties of circulating human influenza viruses [17, 32, 33], so to determine the epitope-specific binding of serum antibodies from ferrets to the epitopes of mAbs 7-1-3 and 11-2-6 on recombinant HA from Vic361(c) a competition enzyme-linked immunosorbent assay (cELISA) was performed. As shown above (Table 2), the aa residues at positions 189 and 193 are critical for mAbs 7-1-3 and 16-3-5, whereas the aa residues at positions 158, 159, 190 and 193 are critical for mAbs 11-2-6, 17-1-1 and 24-3-5. Therefore, mAbs 7-1-3 and 11-2-6 were chosen as representatives for each group that recognize neutralization-sensitive epitopes that are distinct but overlapping at aa residue 193. Fab fragments of the mAbs were used to reduce possible steric hindrance of the binding of serum Abs to neighbouring epitopes.

We tested two or three post-infection ferret antisera (FS) raised against each of Vic361(c and e), egg-propagated A/Texas/50/2012 [Tex50(e)] and Switz13(c and e) virus in competition with the Fabs, using recombinant HA of Vic361(c) as the antigen.

**Fig. 3.**
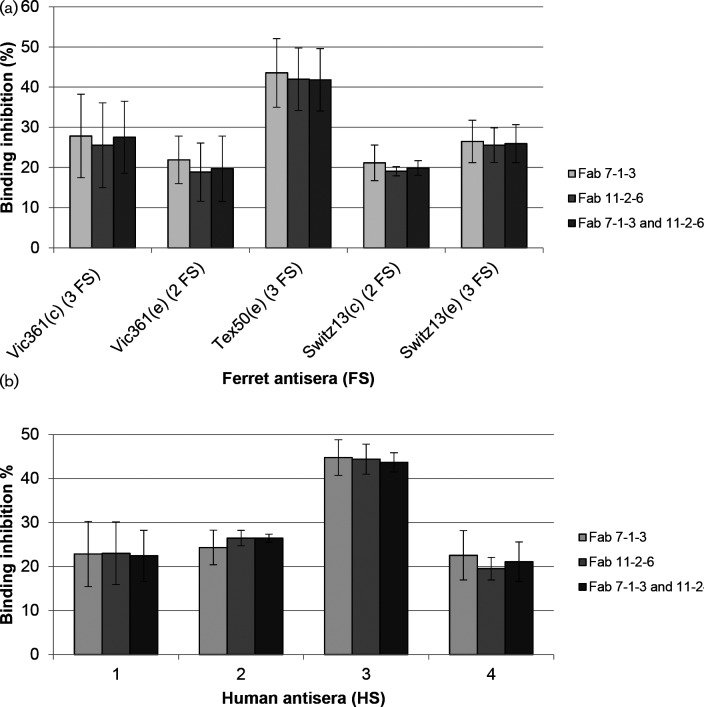
Competition ELISA with post-infection ferret antisera (FS) and human antisera (HS). Ferret antisera obtained after infection with the viruses shown (a) and human antisera collected 7–8 days after vaccination with Vic361-like vaccine virus (b) were tested in competition with Fabs using recombinant HA of Vic361(c) as the antigen. The percentages of binding inhibition by Fabs are shown. The bars show standard deviation.

Fig. 3 shows the average binding inhibition of Abs in FS, through competition with Fabs, as percentages of the binding to HA without prior blocking with Fab. Sera taken from naïve ferrets showed no detectable binding to recombinant HA. The results showed that the presence of either mAb, as Fabs, caused similar reductions in the ability of the FS to bind to the recombinant HA. Moreover, a combination of the Fabs did not increase inhibition of FS Abs. The binding of post-infection FS Abs prepared from ferrets infected with either egg-propagated or cell culture-propagated cultivars of Vic361 and Switz13 was inhibited by 18–28 % by the Fabs, whereas the binding of FS Abs induced by Tex50(e) was inhibited by approximately 42 %. The higher binding inhibition of FS against Tex50(e) suggests that the ferret raised a more focused immune response against the epitopes of Fabs 7-1-3 and 11-2-6, and fewer Abs against other antibody-binding sites of HA. Nevertheless, these results show that all viruses induced FS Abs that recognized epitopes in antigenic site B.

A panel of four human antisera (HS) collected 7–8 days after the vaccination of donors with a Vic361 A(H3N2)-like vaccine virus was also tested in a ELISA with Fab 7-1-3 and 11-2-6 on recombinant HA from Vic361(c). The mAb-blocking assay showed that all HS contained Abs that bind to the epitopes of 7-1-3 and 11-2-6. Both Fabs reduced the binding of each of HS1, 2 and 4 by 19–26 %. The binding of Abs in HS3 was inhibited by approximately 44 %, indicating that HS 3 was more focused and contained a higher proportion of Abs recognizing the epitopes of Fabs 7-1-3 and 11-2-6 than HS 1, 2 and 4. Variations in antibody response in individuals are well recognized [13] and our results show that vaccination with a Vic361-like vaccine induced the production of Abs recognizing neutralization-sensitive epitopes in antigenic site B in all donors.

## Discussion

Influenza viruses change their antigenic properties frequently. However, the range of possible aa substitutions to escape from neutralizing antibodies is restricted, as a fully functional HA molecule needs to be maintained. We generated a panel of mouse mAbs against Vic361 and all were found to be directed against neutralization-sensitive epitopes in antigenic site B, which is thought to be immunodominant in A(H3N2) viruses [14]. The analyses of escape viruses showed that aa substitutions at position 156, 158, 159, 189, 190 or 193 in antigenic site B affect the epitopes of the generated mAbs. Positions 156, 158, 159, 189 and 193 were also identified by Koel e*t al*. as key positions for antigenic changes in viruses circulating between 1968 and 2003 [15]. Our results indicate that changes at positions 156, 158, 159 and 189 affected the epitopes of some mAbs, but not others, whereas substitutions at position 193 had the potential to affect the binding of all mAbs. Hence, aa residues positioned in overlapping epitopes recognized by neutralizing antibodies affect the antigenic properties of more than one epitope. We showed that the identified aa residues also play an important role in A(H3N2) viruses that have been circulating in the last 15 years, with substitutions still occurring, leading to changes in antigenic site B of influenza A(H3N2) viruses. This finding emphasizes the importance of monitoring these key aa residues.

The number of HA glycosylation sites in A(H3N2) viruses has increased since 1968 and it has been shown that oligosaccharides can be associated with shielding of neutralization-sensitive epitopes [9, 22]. Here we show by MS that the potential glycosylation site at position 158 is indeed utilized. This glycosylation has recently been indicated to be very likely using alternative biochemical methods [34]. Our data indicate that this additional glycosylation site, found in most currently circulating clade 3C.2a viruses, modified a neutralization-sensitive epitope in antigenic site B. It is striking that these emerging viruses have escaped from population immunity [16, 17] and we conclude that the addition of the new glycosylation site at aa position 158 altered the antigenic properties of HA. Zost *et al*., using ferret antisera raised against RG viruses with or without the carbohydrate at HA1 position 158, as well as human mAbs isolated from vacinees, adduced that the loss of this carbohydrate in the vaccine virus used recently was the reason for its only moderate effectiveness [34]. Antigenic site B was also implicated to be important in viruses such as Switz13, clade 3C.3a, which have the substitution F159S in HA1. Wild-type Switz13 was recognized less well by all five mAbs used in the HI assay, with the concentrations of mAbs required to inhibit agglutination of RBCs being fourfold or eightfold higher than those required for Vic361(c). RG viruses engineered to encode the substitution F159S in a Vic361(c) background were recognized poorly by five of the seven mAbs used in the HI assays, and RG viruses based on A/Finland/438/2014 (3C.3a) similarly showed that residue 159 influenced binding by four of the five mAbs used in the MN assays. Residue 159 was also implicated in influencing the antigenic properties of clade 3C.3a viruses by Chambers *et al*. [12], who used a post-infection ferret antiserum and a sheep anti-HA antiserum.

Our results show clearly that the panel of mouse mAbs is useful in defining aa changes in HA with the potential to alter antigenicity. Additionally, they can be used to assess epitope-specific immune responses. Using a cELISA with Fab fragments of mouse mAbs 7-1-3 and 11-2-6, recognizing distinct but overlapping neutralization-sensitive epitopes, we showed that post-infection ferret antisera, raised against Vic361(c), Vic361(e), Switz13(c), Switz13(e) or Tex50(e), contain antibodies recognizing the same epitopes in antigenic site B. Interestingly, although the majority of A(H3N2) viruses circulating since 2014 are known to be antigenically distinct from the previously circulating A(H3N2) viruses, we could see that antibodies in ferret antisera, raised against Switz13(c) and Switz13(e), were able to recognize neutralization-sensitive epitopes in antigenic site B of Vic361(c). Our study shows that human antisera from donors vaccinated with a Vic361-derived candidate vaccine virus also contain antibodies against neutralization-sensitive epitopes of HA of Vic361(c). However, the quality of the antiserum is likely to be the result of vaccination and the infection/previous vaccination histories of the individuals, and can perhaps be related to the observations of Wang *et al*., who showed that different sera can contain antibodies with different ranges of specificities [13]. In the light of recent work on antigenic landscaping, a method for the quantitative analysis of antibody-mediated immunity to antigenically variable pathogens [35], the dissection of immune response at the epitope level by techniques such as those utilized in this study could be highly illuminating. This could also provide insights into the phenomenon termed ‘back-boosting’ [35], notably with the use of human mAbs, whereby infection or vaccination can result in the generation of an immune response to influenza viruses or vaccine antigen that the host had been exposed to previously, despite these being antigenically different from the infecting virus or the vaccine antigen administered.

The identification and monitoring of residues responsible for the antigenic drift of circulating A(H3N2) viruses is critical for guiding the selection of virus variants for future vaccine composition. Complementing traditional methods that use post-infection ferret antisera and post-vaccination human antisera for the antigenic analysis of circulating influenza viruses with analysis by mAbs, whether human or mouse, can enhance our understanding of the detailed antigenic properties of emerging influenza viruses.

## Methods

### Viruses and cells

Viruses were propagated either in the allantoic cavity of embryonated hens’ eggs (e) or MDCK-SIAT1 (c) cells, which are Madin–Darby canine kidney cells that overexpress α2,6-sialyltransferase, kindly provided by M. Matrosovich (Institute of Virology, Marburg, Germany) [36]. Cells were maintained in Dulbecco’s modified Eagle’s medium with 10 % (v/v) heat-inactivated foetal calf serum, 100 U ml^−1^ penicillin, 100 µg ml^−1^ streptomycin and 100 µg ml^−1^ G418 sulphate.

### Reverse genetics viruses

Reverse genetics (RG) viruses possessing aa substitutions were generated to identify critical residues in HA of A(H3N2) viruses using the plasmid-based expression system described by Hoffmann *et al*. [37]. HA gene mutations were introduced using QuickChange Site-Directed Mutagenesis kits (Agilent Technologies, USA). Cloning of plasmids and transfection of 293T cells was performed as previously described using either an RG system based on A/Puerto Rico/8/34 or A/WSN/33 [26]. The RG Vic361 viruses had the HA and NA genes from Vic361 and the other six genes from PR8. The RG A/Finland/438/2014 and RG A/Hong Kong/4800/2014 had the HA gene from these viruses, respectively, and all other genes from WSN. Virus recovery was carried out in either embryonated hens’ eggs or MDCK-SIAT1 cells.

### Antisera

Blood samples from volunteers were collected 7–8 days after vaccination with a Vic361-like antigen. Post-infection FS were obtained from stocks held by the Worldwide Influenza Centre, The Francis Crick Institute, London.

### Inactivation of viruses

The A(H3N2) virus A/Victoria/361/2011, propagated either in embryonated hens’ eggs or MDCK-SIAT1 cells, was inactivated for mouse inoculation using beta-propiolactone (BPL) (Ferrak, Germany). The virus was incubated with 0.05 % (v/v) BPL in 100 mM HEPES at 4 °C for 24 h and at 37 °C for 3 h. The virus was pelleted at 100 000 ***g*** for 20 min and resuspended in sterile PBS. The virus concentration was determined using a BCA protein assay kit (Pierce, USA).

### Generation of monoclonal hybridoma cell lines

BALB/c mice were immunized by subcutaneous injection of 30 µg inactivated Vic361 mixed with an equal volume of TitreMax Gold Adjuvant (Sigma-Aldrich, Germany). Boost immunizations of the same dose mixed with adjuvant were applied after 4 and 8 weeks. An intraperitoneal injection of 30 µg inactivated virus in PBS was applied on either days −4 and −3 or days −3 and −2 prior to fusion. Splenocytes were isolated and fused with P3×63Ag8.653 myeloma cells (Sigma-Aldrich, Germany) at a ratio of 2 : 1 in polyethylene glycol 1500 (Roche, Germany). Fused cells were co-cultivated with BALB/c thymocytes in Excell HSF610 medium (Sigma-Aldrich, Germany) containing 20 % (v/v) heat-inactivated FCS, 50 µM 2-mercaptoethanol, 50 U ml^−1^ recombinant murine IL-6, 5.7 µM azaserine and 100 µM hypoxanthine. Aliquots of hybridoma cell clones were incubated in medium without FCS before the supernatant was screened by HI and neutralization assay for secreted neutralizing Abs. Hybridoma cells testing positive were subcloned twice by limiting dilution to obtain monoclonal cell lines and maintained in medium without FCS for the purification of mAbs. Purification of antibody was performed by affinity chromatography using a HiTrap Protein G HP column (GE Healthcare, Sweden). After elution with 0.1M glycine, pH2.7, the mAbs were dialyzed against PBS, pH7.2, and stored at 4 or −20 °C.

### Haemagglutination assay and haemagglutination inhibition (HI) assay

Both haemagglutination and HI assays were performed as described by WHO [38] using 1 % (v/v) guinea pig red blood cells (RBCs) (Marshall BioResources, Hull, UK) in PBS with 20 nM oseltamivir carboxylate (Roche, UK) to circumvent any agglutination of RBCs by the virus neuraminidase [39].

### Microneutralization (MN) assay

The ability of mAbs to neutralize virus was assessed following incubation of mAbs with virus for 1 h and transfer onto confluent monolayers of MDCK-SIAT1 cells in a plaque reduction neutralization assay (PRNA), essentially as described by Lin *et al*. [40].

### Generation of escape viruses

Egg-propagated virus Vic361(e) was incubated with mAb for 1 h at room temperature before inoculation into 11–12-day-old embryonated hens’ eggs to select for mAb escape viruses. Infected eggs were incubated for 48 h at 35 °C before the allantoic fluid was harvested. This selection was repeated and the harvested allantoic fluid tested for escape viruses by HI assay. The HA gene sequences of mAb escape viruses was determined by Sanger sequencing.

### Expression and purification of recombinant A/Victoria/361/2011 HA protein

Recombinant HA from Vic361(c) was generated for a competition enzyme-linked immunosorbent assay . The codon-optimized HA gene was subcloned from plasmid 13AAEYFP_Vic_361_Cell_pMA-RQ, constructed by Life Technologies, USA, into vector pFB-LIC-BSE carrying a trimeric foldon domain and 6x His-tag. The baculovirus, carrying the HA gene, was obtained by using the bac-to-bac expression system (Life Technologies, USA). Insect cells (Sf9) were infected with baculovirus and incubated for 3 days at 28 °C in Sf-900 III serum-free medium (Life Technologies, USA). The supernatant was concentrated and the recombinant HA was purified though HisTalon superflow cartridges (Clontech, USA) and by gel filtration through Superdex-200 10/300 GL (GE Healthcare Life Sciences, USA) with 20 mM HEPES and 150 mM NaCl, pH7.0.

### Antibody cELISA

To determine whether Abs in human (HS) or ferret sera (FS) were binding to the HA epitopes recognized by the generated mouse mAbs, a cELISA was performed. Fab fragments from mouse mAbs were generated using a Pierce Mouse IgG1 Fab and F(ab′)_2_ preparation kit (Thermo Scientific, USA). Recombinant HA from Vic361(c) was incubated on nickel-coated plates (G-Biosciences, USA) at 4 °C overnight and then washed with 0.1 % (v/v) Tween20 in PBS. Fabs in SEA BLOCK blocking buffer (Thermo Scientific, USA) were added. After 2 h incubation, serum, diluted 1 : 800 in blocking buffer, was added for 1 h. Plates were washed and peroxidase-conjugated Abs, recognizing either ferret Abs (SAB3700801, Sigma-Aldrich, Germany) or human Abs (2044–05, Southern Biotech, USA), were added. After washing, 3,3′,5,5′-tetramethylbenzidine (TMB) substrate (Sigma-Aldrich, Germany) was added and, after colour development, the reaction was stopped with 0.1 M H_2_SO_4_. Absorbance was determined by a microplate reader at 450 nm with 620 nm as a reference.

### Mass spectrometry (MS)

Utilization of N-linked glycosylation sites on HA was determined by MS. Cell culture-propagated virus was pelleted through 30 % (w/v) sucrose in PBS at 100 000 ***g*** for 60 min and virus proteins were separated by SDS-PAGE and visualized by Coomassie staining. The excised HA band was incubated in 200 mM ammonium bicarbonate, 50 %(v/v) acetonitrile in water and 10 mM dithiothreitol, and cysteines were then alkylated with 25 mM iodacetamide in 100 mM triethylammonium bicarbonate (pH8.0). After the removal of the alkylation mix, the band was dried by addition of 500 µl acetonitrile, which was removed after the band turned white (indicating dehydration). N-glycans were removed by enzymatic digestion with PNGase F (NEB, USA) in 0.5M sodium phosphate pH7.5 at 37 °C overnight. The solution was removed and the protein digested in-gel with either trypsin or elastase (both 2 µg ml^−1^) at 37 °C overnight. The peptides were separated on an Ultimate 3000 nanoRSLC HPLC followed by tandem MS (MS/MS) on an LTQ Orbitrap Velos Pro (both Thermo Scientific, USA). The data were processed using Proteome Discoverer 2.0 (Thermo Scientific, USA). The identified PNGaseF-mediated deamidation-of-Asn-to-Asp sites were considered to be glycosylation sites if they were located within the motif for N-linked glycosylation and the deamidation-to-amidation ratio was ≥85 %.
